# Empyema of the Antrum of Highmore

**Published:** 1896-10

**Authors:** Frederic C. Cobb

**Affiliations:** Boston, Mass.


ARTICLE VI.

### EMPYEMA OF THE ANTRUM OF HIGHMORE.

BY FREDERIC C. COBB, M. D., BOSTON, MASS.

Empyema of the antrum of Highmore has been for years well known to surgeons both in this country and abroad, but was considered rare on account of a want of comprehension of its symptoms. Jourdain, Deschamps, Cooper, and Desault in the eighteenth century had described it, and Deschamps had even advised catheterization of the antrum by the normal orifice, a piece of advice, however, much more easily given than followed. The antrum was already opened through the canine fossa by Lamourier and Desault, while the alveolar process had been used as a point of approach by Meibomius and Cooper. These cases were of the acute and violent type, and were rare, as they are at the present time. In the records of the Massachusetts General Hospital for the last twenty years I can find but about a dozen cases, and they are all of much the same character. The patient usually entered the hospital complaining of great pain and soreness over the antrum. On examination the whole side of the face was found to be red, swollen, and tender, and perhaps fluctuating at some point below the orbit. The question at first arose as to whether the inflammation was in the antrum or external to it, and in the soft tissues only. Carious teeth were now looked for and extracted when found, and the antrum was washed, the fluid entering at the sinus and passing out of the alveolar opening thus made. The antrum was then packed, or perhaps curetted, and the patient allowed to leave the hospital in a week or ten days, with directions to wash the cavity daily through the alveolar opening. Unfortunately most of these cases occurred so long ago that it is hopeless now to follow their history. No mention of any nasal examination is recorded in any of the older ones, but in most of them carious teeth play an important part. No allusion is made to a possible specific cause, although in the light of our subsequent cases it should, I think, always be considered. Several of these cases occurred in young children from five to seven years of age, and show that where rhinitis persists in childhood antral disease should be thought of. All these cases were, of course, of a violent type and usually not difficult of diagnosis. It is not, however, in the last ten years that the attention of laryngologists has been drawn to a milder form of the same affection. Within the last few years much has been written about the antrum and other sinuses, and light has been thrown upon many obstinate cases of rhinitis which were formerly considered incurable. When we read the reports of the cases I have mentioned it is hard to realize that the disease, as we meet it in laryngological and in surgical clinics, is the same, and that its different symptoms are simply due to the occlusion or patency of the antral orifice. These milder cases we may call latent empyema, or blennorrhœa of the antrum, and instead of the violent symptoms complained of in the form of which I have spoken, are usually signalized by a discharge of a more or less purulent character from one or other nostril, usually accompanied by a foul smell, unlike the smell of ozæna, to the patient himself. This discharge may, of course, be bilaterial if both cavities are involved, but it is not usually so. With this history there arises a consideration of the conditions which may give rise to a unilateral nasal discharge, and these are a foreign body in the nostril, syphilitic disease, and empyema of one of the accessary cavities. On examination of the nose we find the passage clear, thus eliminating foreign bodies from our list. If there is no sign of syphilis, no necrosis of the septum or perforation of its bony wall, we can rule out nasal syphilis with probability. We are then left with an empyema of a sinus, and must make a careful diagnosis as to which accessory cavity is affected. We have to consider in the order of frequency antral, frontal, ethmoid, and sphenoid empyema. Disease of the antrum is by far the most common, probably on account of the connection of that cavity with the teeth, many authors believing that it is ten times as frequent as empyema of any other sinus. Of course the first point to be looked for is the location of the discharge. If pus is seen issuing from under the middle turbinate, the diagnosis is simplified, since the frontal sinus, the anterior ethmoidal, and the antrum have their outlets in that region. The nostril should therefore be carefully cleansed and wiped dry with a pledget of cotton, and then the point of issue of the pus carefully noted. If the pus comes from under the middle turbinate, we next have to differentiate between the antrum, the frontal and anterior ethmoidal cells. This is often a difficult matter, and many expedients have been resorted to in deciding which cavity is affected.

The presence of necrosed bone in the ethmoid region, and the location of the pain, may determine the diagnosis in favor of that cavity. If these signs are wanting, we are left to make a diagnosis between frontal and antral empyema. The location of the pain is here not absolutely reliable, for frontal pain is met with in empyema of the antrum. We may find redness or tenderness over the frontal sinus, and it is recommended to probe the sinus through its opening in the infundibulum. If this fails the anatomical exits of the sinuses may be taken advantage of for the purpose of diagnosis in the following way: As the frontal and ethmoidal have their openings at their lowest parts. while the antral opening is at its highest part, it is evident that the two first sinuses are best drained when the head is in an upright position: but the antrum, when the head is inverted. The patient is therefore directed, after his nostrils have been wiped clear of pus, to put his head between his knees, and if, after a few moments, on assuming the erect position, the nostril is found full of pus, it has probably come from the antrum. The method of transillumination by putting a small electric lamp in the mouth and darkening the room is sometimes useful, as is shown in the cases to-night. Transillumination, however, does not always give certain results, for the variation in the thickness of the walls of the antrum may be considerable, and the skin may be too thick to transmit light well. of these methods fail, recouıse must be had to exploratory puncture by the lower meatus, canine fossa, or alveolar process. The former is preferable, in my opinion, for the following reasons: First, the wall is usually very thin, and, second, there is little or no reactionary inflammation. I have found in perforating through the canine fossa, an immediate swelling of the cheek, which much distorted the face for some days. The passage of the canals was painful, and seemed to increase the swelling. The alveolus is often thick, and therefore more difficult to use as a means of approach. It is, however, on account of its connection with the lowest part of the cavity, and on account of enabling the patient to wash out his own antrum, rapidly becoming the favorite location for puncture. In two of my cases, after an exploratory opening had been made in the lower meatus, carious teeth having their roots in the antrum were extracted and the cavity washed through their sockets, but in both cases the patient preferred to have the washing continued by the lower meatus. If it is decided to puncture by the lower meatus a strong trocar should be introduced below the lower turbinate and turned obliquely outward, as far as the nostril will allow, and then pushed through into the sinus. Often, however, the bone is too thick to permit of this proceeding; it is therefore better, I think, to use a trephine or bur propelled by a dental engine. When the trephine has entered, this is at once felt by the sudden lack of resistance, and it is withdrawn, a canula of the same size fitted to an aspirating syringe introduced, and some of the contents of the antrum removed. For accurate diagnosis this proceeding is better than at once washing out the pus, because the fluid passing over the middle meatus may carry with it pus from the infundibulum, which has not necessarily come from the maxillary sinus. Usually the first washing is accompanied by a very considerable discharge of intensely foul-smelling pus. Ziem has, however, reported very slight amounts of pus from apparently plain cases of empyema, and I have noticed, in two of the cases reported to-night, so little discharge on puncture that the diagnosis seemed to me at the time uncertain, although the result of treatment appears to me proof positive, for the foul discharge improved after the first washing of the antrum and ceased in a short time, although it had lasted many weeks before the puncture. If the pus be too thick to be drawn through an aspirating tube, washing is in reality the only method of ascertaining its presence. In such cases the nostril should be carefully wiped out before washing the antrum. The odor from pus in long-standing antral empyema is almost unbearable, and one can easily understand the distress its constant presence in the nose must cause the patient. Frequently in suspected cases at the time of observation no pus is seen in the middle meatus, and the surgeon is obliged to make the patient lower the head in the manner already described, so as to drain the antrum. Often the patient's history—that pus appears when he lies on the sound side—will give the observer a hint as to the best way of obtaining tbe discharge. I have found a peculiar thin brown mark on the handkerchiefs, a valuable guide to diagnosis which I have not seen described in papers on the subject. This stain has disappeared in the cases treated and cured, and much lessened in those constantly washed. In one case, where nothing could be seen in the nostril, and yet a constant thin brown spot on the handkerchiefs appeared, a probe wound with cotton was inserted under the middle turbinate, and on being removed the stain on the cotton and on the handkerchiefs was found to correspond very well. The antrum was opened through a tooth-socket and washed and cured, the stains disappearing from the handkerchief after a few washings.

The opening was allowed to close eight months ago, and there has been no recurrence of discharge or stains since. Mackenzie has suggested that the pus discharge be examined for bacilli, and this has been done, with as yet no important results as regards diagnosis. The staphylococci pyogenes aurens and albus and citreous, and the pneumococcus of Telamon-Frankel, have been found, the latter of interest since pneumonia is recorded as having followed antral disease. The prognosis in cases as regards tlme of continuance is not of the best, as untreated suppuration lasting many years or through life. It may give rise to many complications directly due to an affection of its neighboring organs or structures, and indirectly to disease of distant organs. Of the neighboring organs the eye is perhaps most frequently affected, and we have iritis, panophthalmitis, narrowing of the field of vision, orbital abscess, and lid abscess, all following empyema of the antrum. Of the skin of the face, facial abscess and pyodermatoses are mentioned, while in ear and throat acute otitis media (from entrance of pus into the Eustachian tube) and pertitonsillar abscess (probably from the passage of pus over the tonsillar region) have been noted. In more distant organs we find reported pneumonia, lung abscess, arthritis, and nephritis. In none of the cases shown by me have any of these complications occurred. As to the question of treatment of empyema of the antrum there is much diversity of opinion. In the fulminant casee there is no question that a wide opening and drainage are imperative. In latent cases, on the other hand, the results of brilliant surgery do not seem to be as gratifying as in some other localities. It goes without saying that the cause must be carefully sought if we are to cure the condition. The most common causes of empyema of this sinus are, I think, in the order of frequency, carious teeth, nasal obstruction, and syphilis. Other less frequent agents of suppuration are foreign bodies in the antrum, such as supernumerary teeth or cotton pledgets introduced into tooth-sockets by the dentist, from thence escaping into the antrum. A rubber drainage-tube has been found as an exciting cause, and had remained some years in the antrum before its discovery and removal. Epidemic influenza seems to have played a prominent part in many cases. In most of my patients the teeth have been an important factor, and in two of them antisyphilitic treatment has decided the diagnosis. I think we may say at present that empyema of the antrum is an obstinate disease, and requires the greatest persistence on the part of both physician and patient. There is, of course, a limit where patience ceases to be a virtue, and mild methods must give place to more energetic surgery, but the fixing of this limit must be dictated by personal experience. Some of my cases were cured in a short time—two or three weeks—some after several months, and others are still under treatment and relapse when the washing is intermitted.

With regard to the location of antral puncture authorities differ, although the weight of opinion seems to be in favor of the alveolar opening. Most of my cases have been washed through the lower meatus, although some have been washed through the canine fossa and the alveolar process. The advisability of a large or small opening into the antrum is still in dispute. Most surgeons believe in the former, while Zeim, with his enormous number of cases, advocates the latter. He, however, makes up for the smallness of the opening by using a powerful pump, which sweeps the antrum out under pressure as it were. Bosworth, on the other hand, advises making an opening in the alveolar process large enough to admit the little finger and thoroughly exploring the antrum. This certainly seems a most rational method of procedure.—*International Dental Journal.*

